# Bayesian statistical learning for big data biology

**DOI:** 10.1007/s12551-019-00499-1

**Published:** 2019-02-07

**Authors:** Christopher Yau, Kieran Campbell

**Affiliations:** 1 Institute of Cancer and Genomic Sciences, University of Birmingham, Birmingham, UK; 2 The Alan Turing Institute, London, UK; 3 Department of Statistics, University of British Columbia, Vancouver, Canada; 4 Department of Molecular Oncology, BC Cancer Agency, Vancouver, Canada

**Keywords:** Bayesian, Computational biology, Statistical modelling

## Abstract

Bayesian statistical learning provides a coherent probabilistic framework for modelling uncertainty in systems. This review describes the theoretical foundations underlying Bayesian statistics and outlines the computational frameworks for implementing Bayesian inference in practice. We then describe the use of Bayesian learning in single-cell biology for the analysis of high-dimensional, large data sets.

## Introduction

Statistics provides a theoretical foundation for rigorous and cohe-rent data analysis by providing a mathematical framework in which to unify models of how data are produced by systems or experiment with techniques to handle uncertainty associated with these processes (Friedman et al. 2001). Whilst there is no single universal statistical approach, one philosophy that has gathered strength in the last 30 years is Bayesian statistical inference (Lindley 1972; Robert 2007; Bernardo and Smith 2009; Gelman et al. 2013). Bayesian statistics offers certain capabilities that enable it to be amenable to a variety of complex statistical applications and constraints, notably in machine learning, where other statistical frameworks would find difficulty. As a consequence, Bayesian approaches are now widely used in a variety of scientific and technological applications including biological research.

In this review, we will examine the fundamental concepts that underpin Bayesian Statistics and consider a concise but otherwise precise overview of the mechanics of applying Bayesian methodology. We will then consider applications of Bayesian techniques in the field of single-cell biology in which technological advances have enabled the high-throughput collection of massive quantities of data that have given us an unprecedented insight into cell function.

## Fundamentals of Bayesian modelling

**Fig. 1:**
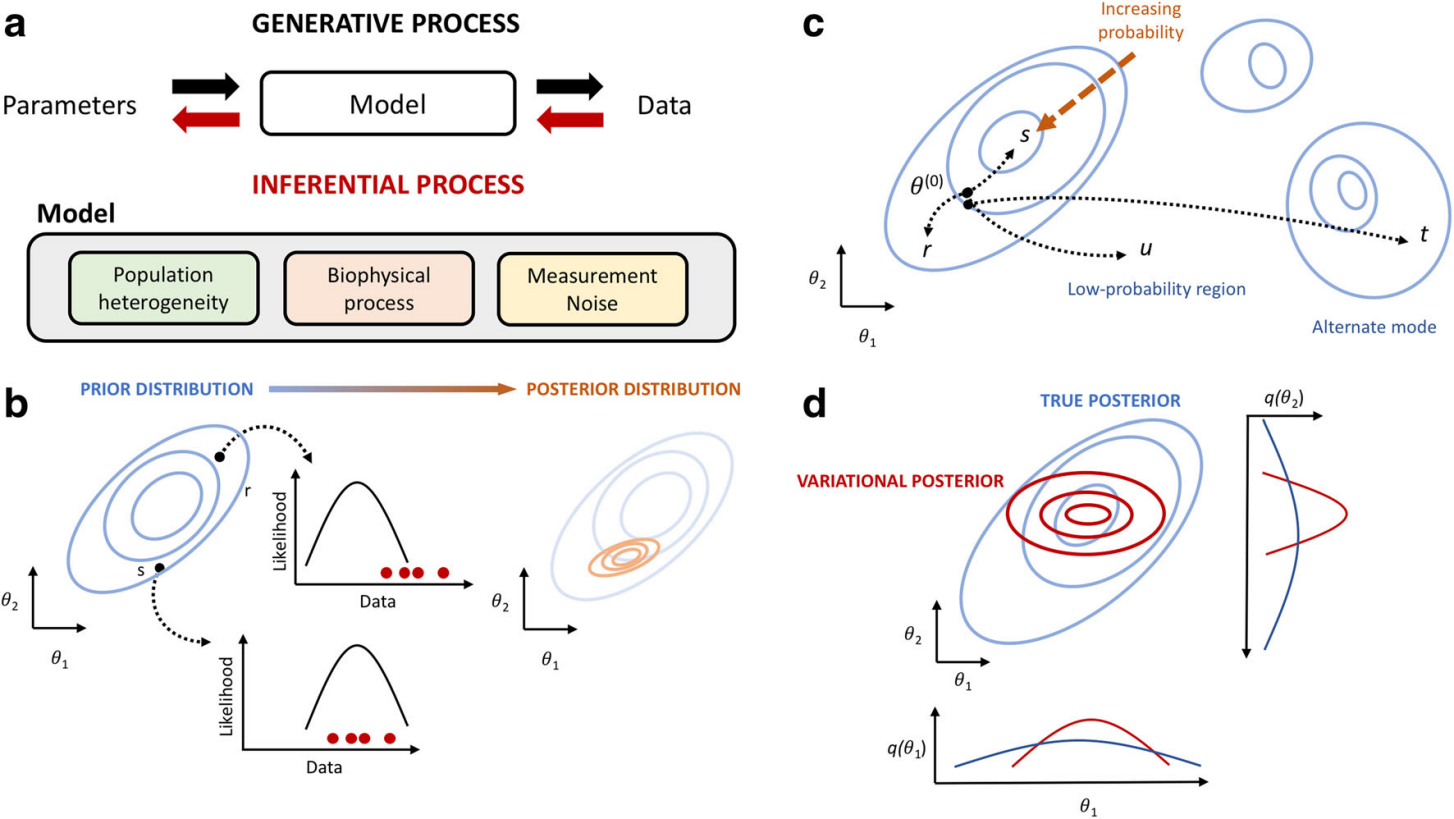
**a** Overview of Bayesian modelling. Data is assumed to be generated by a stochastic model which describes various underlying processes and is specified by some unknown parameters. Bayesian inference seeks to recover those parameters from the observed data. **b** Prior beliefs are expressed as a probability distribution over parameters *𝜃* = (*𝜃*_1_,*𝜃*_2_) which are updated when data is collected via the likelihood function to give a posterior distribution over *𝜃*. **c** Real-world posterior distributions often contain a number of separated high probability regions. An ideal Metropolis-Hastings algorithm would possess a proposal mechanism that allows regular movement between different high-probability regions without the need to tranverse through low-probability intermediate regions. **d** Variational methods build approximations of the true posterior distribution. In this example, a mean-field approximation breaks the dependencies between the parameters (*𝜃*_1_,*𝜃*_2_) so the variational posterior models each dimension separately

Bayesian modelling requires three components (Fig. 1a). The first is *data* (*D*) corresponding to measurements that are taken from the system of interest. Data can range from simple scalar values or, in big data applications, potentially complex structured tuples of multidimensional tensors (Rukat et al. 2017, 2018). The second component is a *generative model* (*M*) which describes a stochastic process by which the observed data arises. The generative model can be mechanistically inspired and based upon real-world physical laws and measurement processes, or may be given by generic statistical models that attempt to describe the dependencies between observed data sources and possibly unseen (latent) factors. Finally, an object of inference (*𝜃*) that we wish to learn about is required. This could be a set of unknown parameters that govern the properties of the generative model which need to be estimated or predictions of future data under alternate conditions.

We can define the *posterior probability* of the object of inference given the observed data in terms of the *likelihood* and *prior probabilities* and the *evidence* via Bayes’ theorem:

1 $$ \underbrace{p(\theta | D, M )}_{\text{Posterior}} = \frac{ \overbrace{p(D | \theta, M )}^{\text{Likelihood}} \times \overbrace{p(\theta )}^{\text{Prior}}} { \underbrace{p(D|M)}_{\text{Marginal Likelihood}}}  $$

What this says is that, given a generative model *M* and data *D*, the posterior probability distribution over the object of inference *𝜃* is given by our prior belief that *𝜃* takes on a certain value, scaled by the likelihood that the generative model under those beliefs would give rise to the data observed (Fig. 1b). The denominator corresponds to a normalising term to ensure that the probability distributions are valid but also describes the *marginal likelihood* of the data under the assumed model. The latter quantity is useful if alternate generative models are available and one can use the marginal likelihood as a means of determining which generative model is likely to be the most consistent with nature. Ratios of marginal likelihoods for different models, say *M*_1_ and *M*_2_, *P*(*D*|*M*_2_)/*P*(*D*|*M*_1_), are known as *Bayes Factors*.

Bayesian statistics can be seen as a coherent system for probability-based belief updating. We begin with some prior knowledge about *𝜃*, we collect data and then we combine the data with our prior beliefs to give our posterior beliefs—what we believe about *𝜃after* seeing data. Importantly, since *𝜃* is an unobserved quantity, Bayesian inference describes our lack of certainty in its value via a probability distribution. If we take an interval of possible values for *𝜃* (a *posterior credible interval*), we can compute the amount of probability mass contained within that interval from the posterior distribution and obtain the probability that the true parameters lie in that region. This interpretation is often considered more natural than the coverage (confidence) intervals used in frequentist-based statistics.

## Bayesian computation

The implementation of Bayesian computation centres on the calculation of the marginal likelihood, *p*(*D*|*M*). This quantity is required to evaluate the posterior probability *p*(*𝜃*|*D*,*M*) and requires a multidimensional integral over all parameters associated with the statistical model. Direct computation is typically intractable, due to the *curse of dimensionality* for any problem of even moderate dimensionality, which results in a combinatorial explosion in the number of configurations that must be summed/integrated over. The challenges are analogous to the computation of the *partition function* in statistic mechanics and Bayesian statisticians have utilised techniques inspired by statistical mechanics to overcome this obstacle in Bayesian computation.

### Monte Carlo methods

*Markov Chain Monte Carlo* (MCMC) simulations (Gilks et al. 1995; Brooks et al. 2011) generate sequences of random numbers such that their long-term statistical properties converge towards the target posterior distribution of interest. The predominant MCMC implementation derives from the Metropolis algorithm formulation in the 1953 paper by Metropolis et al. (1953, whose work was motivated by statistical mechanics applications involving sampling low-energy configurations of complex molecular systems). The technique was later extended in generality by Hastings (1970) to give the *Metropolis-Hastings* (M-H) algorithm. The key insight by Metropolis et al. (1953) was to derive a sampling algorithm which did not require the evaluation of the partition function (marginal likelihood) but only point-wise evaluation of the Boltzmann factors. Given a current configuration of the system *𝜃*, the Metropolis algorithm proceeds by proposing a new state *𝜃*^′^ via any *proposal distribution* and then evaluate the Boltzmann factor exp(−*E*(*𝜃*^′^)/*kT*) at the proposed new state. If the new state results in a lower energy configuration then move to that new state, if it results in a higher energy configuration then choose to move to the new state with a probability which is given by the ratio of the Boltzmann factors: *α* = exp(−(*E*(*𝜃*^′^) − *E*(*𝜃*))/*kT*). By treating the negative logarithm of the unnormalised posterior probability distribution as an energy function, *E*(*𝜃*) = −log *p*(*𝜃*|*D*), the Metropolis algorithm (and its derivatives) has been co-opted by Bayesian statisticians as a means of efficiently performing from complex posterior distributions.

MCMC algorithms provide theoretical guarantees that the stationary distribution of the random number sequences will asymptotically converge to the posterior distribution of interest. The determination of when convergence occurs and designing efficient proposal schemes to enable that convergence to be achieved in the shortest time is highly challenging and remains an area of ongoing research. The critical design choice in the M-H algorithm is the proposal mechanism. If the proposed states are randomly chosen, they are less likely to yield high-probability configurations and will be rejected. If the new states are too similar to the current state, then their probabilities will be similar but the configurations will not be fundamentally different leading to poor exploration of the overall probability space (Fig. 1c). The proposal mechanism must therefore balance the need to search the configuration space globally whilst maintaining a sufficient locality to provide a useful acceptance rate.

A variety of modern MCMC variants now exist (Girolami and Calderhead 2011; Chen et al. 2014; Hoffman and Gelman 2014; Shahbaba et al. 2014). For instance, originally conceived by Duane et al. (Duane et al. 1987) for lattice field theory simulations of quantum chromodynamics, Bayesians have generalised *Hamiltonian Monte Carlo* (HMC) methods (Neal et al. 2011) which exploit geometric information to greatly increase the sampling efficiency of MCMC algorithms. Whilst standard M-H algorithms can be described as a *propose-and-check* approach, HMC biases proposals along trajectories that are likely to lead to high-probability configurations. Probabilistic programming languages such as Stan (Carpenter et al. 2016) and PyMC3 (Salvatier et al. 2016) contain prebuilt implementations of HMC and variants freeing modellers from many of the detailed requirements of building HMC algorithms.

### Variational methods

The computational requirements of MCMC methods can be prohibitive in applications that involve large, high-dimensional data sets or complex models. As the dimensionality of *𝜃* increases, the convergence complexity of MCMC algorithms also increases when sampling from high-dimensional posteriors (Mengersen et al. 1999; Rajaratnam and Sparks 2015). An alternative is to abandon the theoretical guarantees of MCMC methods and to construct analytically tractable approximations *q*_*ν*_(*𝜃*|*D*) to the true posterior distribution *p*(*𝜃*|*D*)—this is the motivation underlying *Variational Bayesian* methods (Blei et al. 2017).

In the construction of variational approximations, it is typical to assume that the approximating distribution has a simplified structure (Fig. 1d). The frequently used *mean-field* approximation assumes a fully factorisable form of the approximate posterior, $q_{\nu }(\theta |D) = {\prod }_{t = 1}^{T} q_{\nu }^{(t)}(\theta _{t}|D)$ where the dependencies between the different elements of *𝜃* are uncoupled and each factor $q_{\nu }^{(t)}$ is typically given by a simple distribution (e.g. Gaussian, Gamma). If the approximating distribution *q*_*ν*_ is parameterised by *ν*, the variational approach seeks to optimise these *variational parameters* to minimise the difference—measured using the Kullback-Leibler (KL) divergence—between the true and approximate posterior distributions. Therefore, unlike Monte Carlo methods which use stochastic sampling, variational methods transform the inference problem into an optimisation task. The latter means that assessing the convergence of variational methods is relatively straightforward and typically requires significantly less time for complex models than MCMC approaches.

Classic variational algorithms used analytically derived optimisation steps (coordinate ascent VI) but, more recently, stochastic variational inference (SVI) methods employ stochastic gradient descent algorithms instead (Hoffman et al. 2013; Titsias and Lázaro-Gredilla 2014). SVI uses cheap to compute, “noisy” estimates of natural gradients based on a subset of data points instead of the true gradients which require a pass through all data points. This exploits the fact that the expected value of these noisy gradients is equal to the true gradient and so convergence of the SVI algorithm can be guaranteed under certain conditions. As a consequence, SVI allows the application of variational methods to a wider class of models and by operating on *mini-batches* of data in each optimisation step provides substantial speed-ups in large data settings.

*Amortised variational inference* uses *inference networks* within variational inference algorithms for latent variable models—where each data item is associated with its own set of parameters (Zhang et al. 2018). In such situations, a typical variational approximation would result in each data item also being associated with its own compliment of variational parameters; thus, with larger data sets, there would be an increase in the number of variational parameters to optimise. Inference networks replace these *local* variational parameters with a single set of global parameters associated with a neural network. The goal is to use variational inference to optimise the parameters associated with this neural network and to use the optimised network to *predict* the local variational parameters. Inference networks therefore offer another layer of approximation that breaks the dependency between the computational requirements of the variational inference algorithm and the size of the data set.

Whilst the accuracy of variational approximations is often impossible to quantify (Yao et al. 2018), they provide the current mainstay inferential approach for high-dimensional Bayesian modelling. Current machine learning development libraries, such as TensorFlow (Abadi et al. 2016) and PyTorch (Paszke et al. 2017), provide a substantial body of tools for the construction of neural networks, and optimisation algorithms for the implementation of variational inference algorithms.

## Bayesian applications in single-cell biology

The recent availability of a plethora of relatively low-cost experimental methods and protocols for high-throughput screening of individual cells has lead to an explosion in single-cell biological data (Theillet 1998). A single-cell experiment can generate data that is both high-dimensional and large in sample size with recent studies involving single-cell RNA sequencing routinely able to produce cell numbers on the order of 10^5^ cells measuring 10^3^ − 10^4^ genes. International endeavours, such as The Human Cell Atlas (HCA) project (Regev et al. 2017), will seek to catalogue and sequence all known human cell types in the coming years.

The benefit of single-cell measurements is to remove the averaging effect when measurements are taken on populations of cells which can obscure important stochastic dynamics operating in individual cells. However, the challenge when working with single cells is the inherent sensitivity of cells to physical manipulation and the difficulties of robustly measuring minuscule quantities of potentially unstable molecules, e.g. RNA. Consequently, single-cell data from any technical platform is inherently noisy, contains various levels of missingness and may harbour many sources of bias—all of which could have both a biological or technical origin (Stegle et al. 2015; Poirion et al. 2016). Probabilistic modelling of single-cell data, based on a Bayesian framework, provides a coherent strategy for encapsulating these complexities.

### Differential expression

**Fig. 2:**
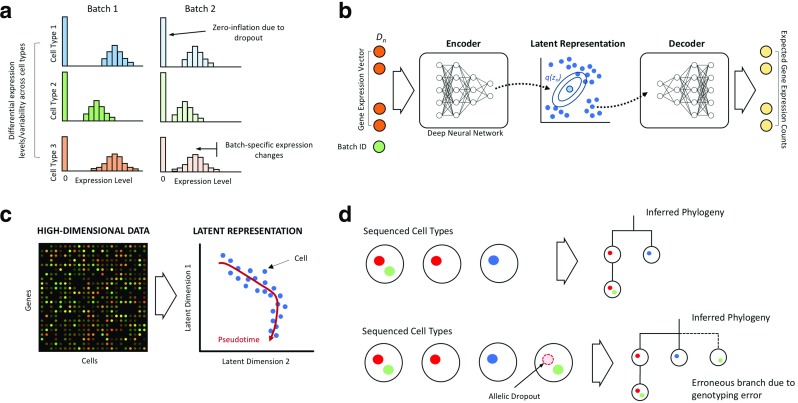
**a** Single-cell differential expression analysis aims to identify differences in expression level and variability between cell types. Confounding effects such as dropout and batch effects must be accounted for in order to avoid false conclusions. **b** Variational autoencoders use deep neural networks to *encode* input expression data vectors into low-dimensional latent representations whilst simultaneously learning *decoders* that can generate realistic expression data from these latent representations. **c** Pseudotemporal model aims to identify latent uni-dimensional representations that correspond to physical time variation from high-dimensional cross-sectional single-cell data. **d** Probabilistic approaches to tumour phylogeny inference are essential in the presence of sequencing noise since genotyping errors can lead to uncertainties in phylogenetic reconstruction. Here, the presence of allelic dropout leading to genotyping error in a single-cell type could lead to alternate phylogenetic histories and different interpretations of the importance of acquired mutations

*Differential expression* (DE) aims to identify genes that are up- or downregulated between cell types (Fig. 2a). Whilst standard frequentist-based hypothesis testing procedures can be employed, Bayesian DE alternatives offer certain benefits. Kharcenko et al. (2014) introduced a generative model that includes drop-outs for differential expression analysis. Dropouts are frequent occurrences in single-cell expression data due to the low quantities of mRNA involved which means the presence of some transcripts cannot always be reliably detected by sequencing—the result is a zero expression measurement for cells that might actually be expressing a gene at a low level. BASiCS (Bayesian Analysis of Single-Cell Sequencing data (Vallejos et al. 2015; Vallejos et al. 2016)) jointly models highly variable genes and differential expression between cell populations which allows it to detect *differential variability*—an effect often masked by both standard differential expression methods. A related analysis is identifying differential splicing in which exon usage varies between cells or cell populations. Differential splicing can be difficult to detect in single-cell RNA-seq data due to amplification biases, shallow read depths, and other technical artefacts. To solve this, the Bayesian method BRIE (Bayesian regression for isoform estimation (Huang and Sanguinetti 2017)) leverages sequence-derived features as an informative prior distribution in a hierarchical model to greatly increase the accuracy of inference.

### Deep learning representations

One particular analytical problem is the identification of latent structure within these high-dimensional data sets in order to understand the underlying fundamental biological processes. Ideas inspired from *deep learning* (LeCun et al. 2015) have recently emerged in single-cell biology as a means of extracting low-dimensional representations from high-dimensional data (Ding et al. 2018; Lopez et al. 2018). For instance, scVI (Lopez et al. 2018) uses a hierarchical Bayesian model—a variational autoencoder (Kingma and Welling 2013), incorporating deep neural networks and stochastic variational inference stochastic optimisation to aggregate information across similar cells and genes whilst simultaneously adjusting for batch effects and lack of measurement sensitivity (Fig. 2b). The benefit of the deep neural networks is that the functional relationship between the measured gene expression and the latent representations does not need to be prespecified by the modeller and scVI is able to exploit the vast array of data available to *learn* these relationships from the data itself. Implementations using modern machine learning development frameworks allow a vast array of high-performance computational machinery (such as graphics processing units) to be exploited permitting methods such as scVI to make short work of data sets involving millions of cells.

### Temporal modelling

High-throughput single-cell molecular technologies provide an instantaneous measurement of the molecular state of individual cells. Genuine time series measurements of individual cells undergoing dynamic processes, such as differentiation or cell cycle, are difficult due to the inherently destructive nature of the measurement process and asynchronicity of cellular progression. To circumvent this, analytical methods have been developed that use a cross-sectional “snapshot” of cells’ gene expression to assign a *pseudotime* to each cell—a surrogate measure of progression through the process of interest (Fig. 2c). Downstream analyses such as differential expression (Campbell and Yau 2017b; Sander et al. 2017) can then be performed using the pseudo times in lieu of physical time information.

A majority of Bayesian pseudotime inference methods build upon the Gaussian Process Latent Variable (GPLVM) framework. The first model for single-cell RNA-seq was DeLorean (Reid and Wernisch 2016) that uses a Matern_3/2_ kernel with a Gaussian likelihood on suitably log-transformed data. DeLorean uses the probabilistic programming language Stan (Carpenter et al. 2016) for inference that performs an adaptive version of Hamiltonian Monte Carlo. This was recently reimplemented in the method GrandPrix (Ahmed et al. 2019) with fast inducing point variational inference implemented in the GPFlow framework (Matthews et al. 2017) to achieve order-of-magnitude faster inference. A related model is the PseudoGP framework (Campbell and Yau 2016) that uses the posterior distributions from probabilistic pseudotime to quantify the uncertainty in downstream analyses such as differential expression. Branching differentiation processes can also be modelled using Gaussian processes (Boukouvalas et al. 2018; Penfold et al. 2018).

Further, Bayesian pseudotime methods have been developed based on dimensionality reduction techniques other than GPLVM. A popular class of these are factor analysis models that seek a probabilistic mapping from the latent space (pseudotimes) through a linear or parametric nonlinear function. Such an approach was successfully applied in the Ouija framework (Campbell and Yau 2018) that uses a sigmoidal mapping to learn pseudotimes from small marker gene panels along with interpretable parameters corresponding to activation times of genes. A related model is MFA (Campbell and Yau 2017a) that implements a mixture of linear factor analysers to infer bifurcations from single-cell gene expression data, using MCMC sampling for inference. Finally, a Bayesian variant of unidimensional scaling (BUDS, Nguyen and Holmes 2017) has been proposed for ordering single cell with an emphasis on visualising uncertainty.

### Tumour evolution

Bayesian approaches have also been developed for single-cell–based modelling of cancer evolution (Zafar et al. 2018; Goh et al. 2019). Here, the data corresponds to genome sequences of tumour samples and the unobserved object of inference is the evolutionary tree relating the different cancer cell populations within the tumour (Yuan et al. 2015; Roth et al. 2016) or a mutation tree representing the partial (temporal) order of the mutation events (Jahn et al. 2016; Ross and Markowetz 2016). Since an arbitrary number of evolutionary mechanisms may be possible, the information included in the priors help to *regularise* the inferential problem to make it tractable by limiting the space of possible evolutionary trajectories. Uncertainty propagation is also of specific help in this problem. Allelic dropout in single-cell sequencing can cause mutations to become undetected and lead to errors in the genotyping of individual cells. Errors in cellular mutation profiles could fundamentally alter the inferred evolutionary trees hence joint modelling of sequencing errors and evolutionary trajectories is critical (Fig. 2d).

## Discussion

Bayesian methodology is a conceptually natural approach to apply to biological research applications. Modern probabilistic programming language environments for Bayesian computation have further facilitated its application by providing interfaces for specifying potentially highly complex models even for non-experts. This review has described the underlying theoretical framework as well as the computational techniques required to implement Bayesian modelling with a focus on applications in single-cell biology. Nonetheless, further research into improved and faster Bayesian computation techniques for big data biology is required. Despite its strong theoretical foundations, Bayesian approaches are still relatively underused in biological sciences. Bayesian modelling requires considerable thought to be given to the constitution of the generative models and the specification of prior beliefs. Probabilistic programming languages have simplified model development by allowing users to focus on model specification rather than the computational implementations but there remains a considerable “art” to designing good models and expertise is gained through experience. Research to develop more *automatic* tools for Bayesian model specification would be beneficial. Posterior uncertainty characterisation intrinsically means that there is no “right answer” in Bayesian modelling—only a distribution over possibilities. Probabilistic outcomes can be difficult to interpret even for seasoned experts and non-experts may find such summaries challenging to palate. Finally, in high-dimensional, large data settings, recent computational advances have made Bayesian inference feasible for increasingly larger problems but often remains more computationally taxing than alternative approaches that might forego uncertainty characterisation for point estimation. However, as described in many of the single-cell applications, without formal uncertainty modelling, erroneous inferences can be made in the presence of confounding factors or noisy/missing data.
